# Comprehensive Molecular Analysis of *DMD* Gene Increases the Diagnostic Value of Dystrophinopathies: A Pilot Study in a Southern Italy Cohort of Patients

**DOI:** 10.3390/diagnostics11101910

**Published:** 2021-10-15

**Authors:** Fatima Domenica Elisa De Palma, Marcella Nunziato, Valeria D’Argenio, Maria Savarese, Gabriella Esposito, Francesco Salvatore

**Affiliations:** 1 CEINGE-Biotecnologie Avanzate s.c.ar.l., 80145 Naples, Italy; depalma@ceinge.unina.it (F.D.E.D.P.); nunziato@ceinge.unina.it (M.N.); dargenio@ceinge.unina.it (V.D.); savaresem@ceinge.unina.it (M.S.); 2 Department of Molecular Medicine and Medical Biotechnologies, University of Naples Federico II, 80131 Naples, Italy; 3 Department of Human Sciences and Quality of Life Promotion, San Raffaele Open University, Via di Val Cannuta 247, 00166 Roma, Italy

**Keywords:** DMD/BMD, Duchenne/Becker muscular dystrophy, dystrophin, next-generation sequencing, MLPA, multiplex PCR, Sanger sequencing, molecular diagnostics

## Abstract

Duchenne/Becker muscular dystrophy (DMD/BMD) is an X-linked neuromuscular disease due to pathogenic sequence variations in the dystrophin (*DMD)* gene, one of the largest human genes. More than 70% of *DMD* gene defects result from genomic rearrangements principally leading to large deletions, while the remaining are small nucleotide variants, including nonsense and missense variants, small insertions/deletions or splicing alterations. Considering the large size of the gene and the wide mutational spectrum, the comprehensive molecular diagnosis of DMD/BMD is complex and may require several laboratory methods, thus increasing the time and costs of the analysis. In an attempt to simplify DMD/BMD molecular diagnosis workflow, we tested an NGS method suitable for the detection of all the different types of genomic variations that may affect the *DMD* gene. Forty previously analyzed patients were enrolled in this study and re-analyzed using the next generation sequencing (NGS)-based single-step procedure. The NGS results were compared with those from multiplex ligation-dependent probe amplification (MLPA)/multiplex PCR and/or Sanger sequencing. Most of the previously identified deleted/duplicated exons and point mutations were confirmed by NGS and 1 more pathogenic point mutation (a nonsense variant) was identified. Our results show that this NGS-based strategy overcomes limitations of traditionally used methods and is easily transferable to routine diagnostic procedures, thereby increasing the diagnostic power of *DMD* molecular analysis.

## 1. Introduction

Duchenne muscular dystrophy (DMD—OMIM number 310200), as well as the allelic Becker form (BMD—OMIM number 310376), is a lethal, rapidly progressive neuromuscular disease, whose characteristic trait is the degeneration of skeletal, smooth, and cardiac muscles leading to progressive muscle fiber damage and loss of muscle function [1,2,3]. Elevated serum level of creatine kinase is early hallmark of the disease that begins in childhood (onset at four or five years of age) and estimated incidence, for the severe DMD form, is of about 1:3300 males; females are usually asymptomatic, though a small percentage may show a mild disease-related phenotype [2,3,4,5]. DMD/BMD is an X-linked recessive disease caused by sequence alterations occurring in the *DMD* gene (OMIM *300377) encoding the dystrophin protein [6,7]. Dystrophin is an important anchor protein that plays a role in anchoring the cytoskeleton to the plasma membrane through F-actin. In the absence of dystrophin, muscle cells become more permeable; the extracellular matrix enters the cells leading to the destruction and progressive death of these cells that are replaced by adipose tissue [8].

*DMD* is one of the largest known human genes; it encompasses 79 exons, spanning approximately 2.4 Mb [9]. In addition to its large size, *DMD* is featured by a complex mutational spectrum, since more than 7000 pathogenic variants are known to date. About 70% of *DMD* mutations result from genomic rearrangements (GRs) that lead mainly to large deletions and to a lesser extent to duplications involving one or more gene exons [10]. The remaining mutations are small nucleotide variants (SNVs), including nonsense and missense variants, small insertions/deletions (INDELs) or splicing alterations, which can occur anywhere along the gene [1,6,11,12]. Thus, *DMD* molecular analysis is complex and requires multiple analytical steps to reach enough diagnostic sensitivity. However, confirmation of the DMD/BMD clinical suspicion should be as quick as possible to ensure appropriate patient care, carrier identification, family planning, prenatal diagnosis and, most importantly, prompt access to personalized treatment [13,14].

Next generation sequencing (NGS)-based approaches are currently used for routine molecular diagnostics [6,15,16]. Indeed, these approaches have shown their reliability and accuracy in the analysis of single disease-causing genes, panels of genes related to a disease of interest, and the whole exome/genome [17,18,19,20,21,22,23]. Consequently, NGS is redefining the standards for the detection of SNVs related to the onset of human diseases. 

Recently, it has been proposed that the high sequence coverage, unusual of NGS applications, may be used to estimate the presence of copy number variations (CNVs) often associated with GRs [24,25]. In this way, NGS may be able to allow, in a single analytic procedure, the complete diagnosis of a disease of interest by detecting both SNVs and GRs [26]. In this contest, we have recently shown that an NGS-based method, coupled with specific bioinformatic tools, was able to identify large heterozygous deletions/duplications in different disease-associated genes [23,27].

Here, we aimed to verify if the same analytic strategy could be effective to improve molecular diagnosis of DMD/BMD. For this purpose, we tested an NGS method suitable for the comprehensive detection of all the different types of genomic variants affecting the *DMD* gene. Our results show that the proposed strategy overcomes the limitations of traditionally used methods and is easily transferable to routine diagnostic procedures.

## 2. Materials and Methods

### 2.1. Study Population and DNA Samples

Forty DNA samples were selected among those who underwent a *DMD* molecular analysis at the CEINGE molecular diagnostic core lab. In particular, 31 DMD/BMD patients (males, aged from 3 to 61 y), and 9 DMD carriers (females, aged from 13 to 62 y) were included in this study. All patients gave their written informed consent to the molecular analysis. The study was conducted according to the guidelines of the Declaration of Helsinki, and approved by the Ethics Committee of University of Naples Federico II (protocol code 77/21, 26 March 2021).

To assess the accuracy in mutations detection of the proposed NGS-based method, patients were selected among those carrying different *DMD* mutations, spanning all over the *DMD* gene sequence. In particular, 4 carried point mutations identified by Sanger sequencing; 32 patients harbored large deletions/duplications identified by multiplex PCR and/or MLPA analysis; and 4 resulted in a wild type after the multiplex PCR and/or MLPA analysis. NGS-*DMD* screening was carried out blind.

### 2.2. DMD Gene Traditional Molecular Analysis

Genomic DNAs were obtained by peripheral blood samples in EDTA by using standard procedures. For CNVs detection, quantitative fluorescence multiplex PCR and/or MLPA analyses were carried out. In particular, 4 multiplex PCR reactions/patient were performed on a 2700 thermal cycler (Applied Biosystems Inc., Foster City, CA, USA) to analyze the 24 *DMD* exons corresponding to CNVs hot-spot, using labeled primers and an internal standard reference as a control to be added to each multiplex PCR. The obtained PCR products were separated by capillary gel electrophoresis on the ABI PRISM 3130 XL genetic analyzer (Applied Biosystems, Foster City, CA, USA), as previously reported [28]. The MLPA test was performed to screen all the dystrophin gene exons using the SALSA MLPA probe sets P034/P035 (MRC-Holland, Amsterdam, the Netherlands), according to the manufacturer’s instructions. Ligation and amplification steps were performed using a 2700 thermal cycler (Applied Biosystems Inc., Foster City, CA, USA). Next, all the amplified fragments were separated using capillary electrophoresis on an ABI PRISM 3130 XL genetic analyzer and data analysis was carried out using the Coffalyser software (MRC-Holland, Amsterdam, the Netherlands). 

Sanger sequencing was used to detect point mutations already identified within the family. PCR amplification was carried out on a 2700 Thermal Cycler (Applied Biosystems Inc., Foster City, CA, USA) to specifically amplify the *DMD* exon carrying the pathogenic mutation. Next, direct sequencing was performed using an ABI 3100 capillary sequencer (Applied Biosystems Inc., Foster City, CA, USA). Sanger electropherograms were visualized using the SeqMan tool (DNASTAR, Inc., Madison, WI, USA).

### 2.3. NGS Analysis

All the selected DNA samples, already analyzed as described above, were quantified by using the Thermo Scientific™ NanoDrop spectrophotometer, and quality-assessed on agarose gel (0.8% agarose and 0.1 μg/ml ethidium bromide). Next, DNA indexed libraries were prepared using Multiplicom’s *DMD* MASTR assay protocol (Multiplicom, Niel, Belgium), following the manufacturer’s instructions. Briefly, for each sample, 200 ng of genomic DNA were used as input. All the *DMD* target regions were amplified in 4 separate multiplex PCR amplification reactions. A second round of amplification (Universal PCR) was performed to univocally tag all the obtained amplicons from the same DNA sample with a specific barcode sequence (INDEX) and the p5-p7 adaptors, required for sample multiplexing and the downstream sequencing reactions. Each indexed amplicon library was then purified using the Agencourt AMPure XP beads (Beckman Coulter, Inc., Brea, CA, USA), and verified for quality using the Agilent 2100 Bioanalyzer with a DNA 1000 LabChip (Agilent Technologies, Santa Clara, CA, USA). All the 40 indexed libraries were pooled in equimolar amount and sequenced on the MiSeq system (Illumina Inc., San Diego, CA, USA) using the Illumina Reagent Kit V2 (500 cycles PE, 2 × 250 bp). Sanger sequencing was used to confirm identified causative point mutations or doubtful variants.

### 2.4. NGS Sequence Data Analysis

The downstream sequencing data analysis was carried out using 2 different bioinformatic software: (i) the Sequence Pilot software (version 4.2, JSI Medical Systems GmbH, Kippenheim, Germany) used to detect point mutations; and (ii) the Sophia DDM® software (version 4.8.1.3, Sophia Genetics SA, Saint Sulpice, Switzerland), used to verify the presence of point mutations and identify large GRs (LGRs). 

In particular, to detect possible SNVs and small INDELs in the sequenced samples, the SeqNext module of the JSI SeqPilot maps all the obtained sequencing reads against the *DMD* reference sequence (ENSG00000198947 gene reference and ENST00000357033 transcript from Ensemble database). Reads corresponding to each sample were sorted according to their INDEX and variants were called only if their frequency was more than 10% considering the combined reads, excluding homopolymers (repetition of 6 or more identical nucleotides), according to the manufacturer’s instructions and the default parameters suggested by the JSI SeqPilot software. All the detected variants were annotated and classified according to their biological and clinical significance using different databases, such as Ensemble (http://www.ensembl.org, accessed on 12 October 2021), ClinVar (https://www.ncbi.nlm.nih.gov/clinvar/, accessed on 12 October 2021), and Varsome (https://varsome.com/, accessed on 12 October 2021).

The Sophia Genetics DDM^®^ software, in addition to call SNVs, can deduce the sex of each patient based on their homozygous and heterozygous status. Further, the software implements an algorithm able to automatically select, based on coverage similarities, a set of reference samples among those sequenced in the same run. Then, using these reference samples, the coverage is normalized by sample and by the target region, and CNVs calling is performed using a hidden-Markov-model algorithm [11]. Sophia DDM software classifies all samples into rejected, medium, and low noise, based on the residual coverage noise after the step of normalization and CNVs calling. No CNV results were reported for the rejected samples, but plots of the coverage profile for those samples were still included for illustration purposes.

**Figure 1:**
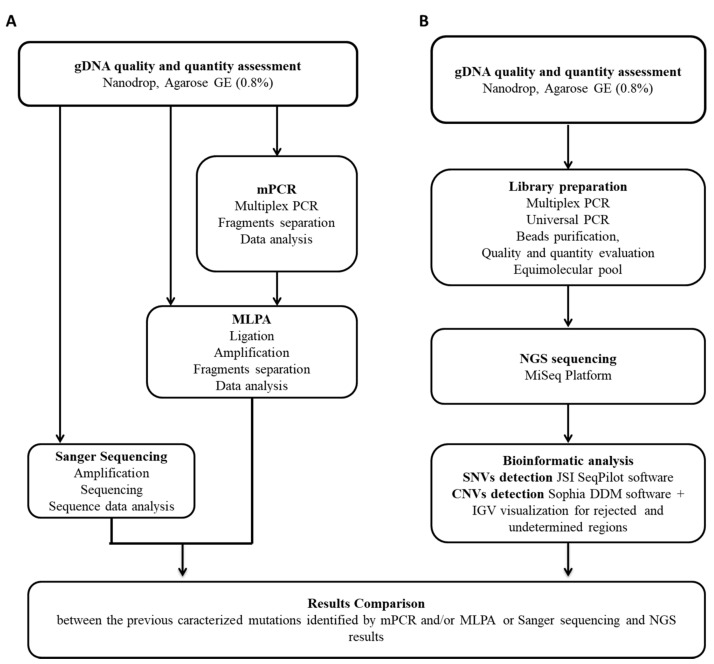
Summary of the analytic workflow. After genomic DNA quality and quantity assessment, each sample has been analyzed according to traditional molecular techniques (**A**) and NGS (**B**). In particular, MLPA and/or Sanger sequencing were carried out to detect *DMD* pathogenic mutations (**A**). The same samples were analyzed blindly by NGS (**B**). DNA libraries were prepared with an amplicon-based protocol for each study subject. Obtained libraries (corresponding to 40 individual samples) were sequenced in one sequencing run using the MiSeq system. NGS sequence data analysis was carried out using two different pipelines. Finally, NGS results were compared to those obtained with conventional diagnostic procedures.

In not-rejected samples, individual target regions were classified into three categories: high-confidence, medium-confidence, and undetermined. The performance of the CNV module is higher for longer CNVs than for shorter CNVs and higher for deletions than for duplications. In most cases, single-amplicon duplications would not be missed, but labeled as “undetermined”. However, it is recommended to re-test, for validation purposes, all the GRs found using an independent test, i.e., MLPA. The entire workflow is summarized in Figure 1.

## 3. Results

The 40 patients included in this study were analyzed following the routine diagnostic procedure for *DMD* (multiplex PCR and/or MLPA and/or Sanger sequencing), as described under the methods section. Moreover, they were analyzed blindly with the NGS-based strategy described above to assess its performance in diagnostic settings. 

### 3.1. NGS Sequence Coverage

Each amplicon library was successfully analyzed as described under Materials and Methods. In total, we obtained 8,706,134 read pairs with 90.60% (7,887,326 reads) of mapped reads. The 90.23% of reads obtained were on target, while 1.12% and 8.65% were respectively “off-target” and unmapped reads. On target coverage distribution per sample is reported in Supplementary Table S1.

### 3.2. Point Mutation Identification

**Figure 2:**
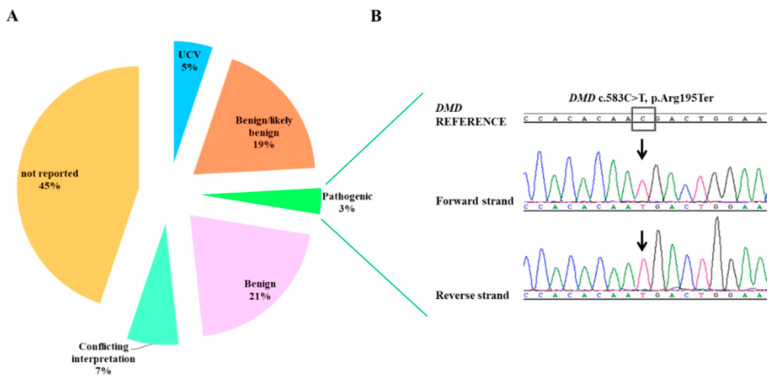
Single nucleotide variants (SNVs) identified in the study group. (**A**) Pie chart illustrating the distribution (%) of the SNVs detected by NGS and classified according to their clinical significance by ClinVar Database; 3% of them corresponds to pathogenic mutations. (**B**) The c.583C>T (p.Arg195*) hemizygous mutation was found in one male subject by NGS and confirmed by Sanger sequencing as shown in the electropherogram assembled with the reference sequence ENST00000357033.8 (NM_000109; NP_000100). The figure highlights the presence of the variant in the forward and reverse strand respectively, as indicated by the arrows. DMD, Duchenne muscular dystrophy; NGS, next generation sequencing; UCV, uncertain significance variant.

Fifty-eight total variants, scattered along the whole *DMD* gene, were detected. Among these, 39 (67%) were intronic and 19 (33%) were exonic variants. Among the 19 coding sequence variants, 11 (58%) were missense, 3 (16%) were nonsense variants, 3 (16%) were synonymous, and 2 (10%) were frameshift variants. All the detected variants were classified according to their clinical significance using the ClinVar database and/or American College of Medical Genetics (ACMG) classification (Figure 2A, Table 1). 

As reported in Table 1, 3 pathogenic nonsense variants were detected: 2 were already identified by the previous Sanger sequencing analysis (c.3259C>T, p.Gln1087* and c.2414C>G, p.Ser805*), the other one (c.583C>T, p.Arg195*) was detected in a DMD patient (ID-5) who resulted wild type after the previous multiplex PCR and MLPA analysis.

Sanger sequencing was carried out to confirm this NGS data (Figure 2B). In addition, two frameshift mutations causing a premature stop codon were also identified confirming previous Sanger results.

### 3.3. CNVs Detection

CNVs analysis was performed on the whole cohort of samples by the Sophia Genetics software (Table 1). The sex of each sample was successfully identified by the software. Twelve samples, automatically selected by the software, were used as control references. One hundred forty duplicated target regions and 110 deleted target regions were achieved. The average coverage per target region was 1623X, with average residual noise of 0.0913. 

Of the 40 analyzed samples, 24 were classified as samples with low-noise, 12 with medium-noise, and only 4 were rejected from the CNV analysis due to the background noise level (Figure S1, Table S2). Five samples showed some undetermined regions, probably corresponding to the presence of duplications in a single amplicon. Indeed, comparing results of the previous multiplex PCR/MLPA with Sophia software results, all the undetermined regions resulted to be duplicated exons (Figure S2, Table 2). Three out 4 rejected samples had deletions that therefore escaped to the Sophia software analysis. However, despite the software considering them rejected, it is easy to recognize the presence of deletions through a virtual inspection with the IGV software (see also Supplementary Materials, Figure S1).

**Figure 3:**
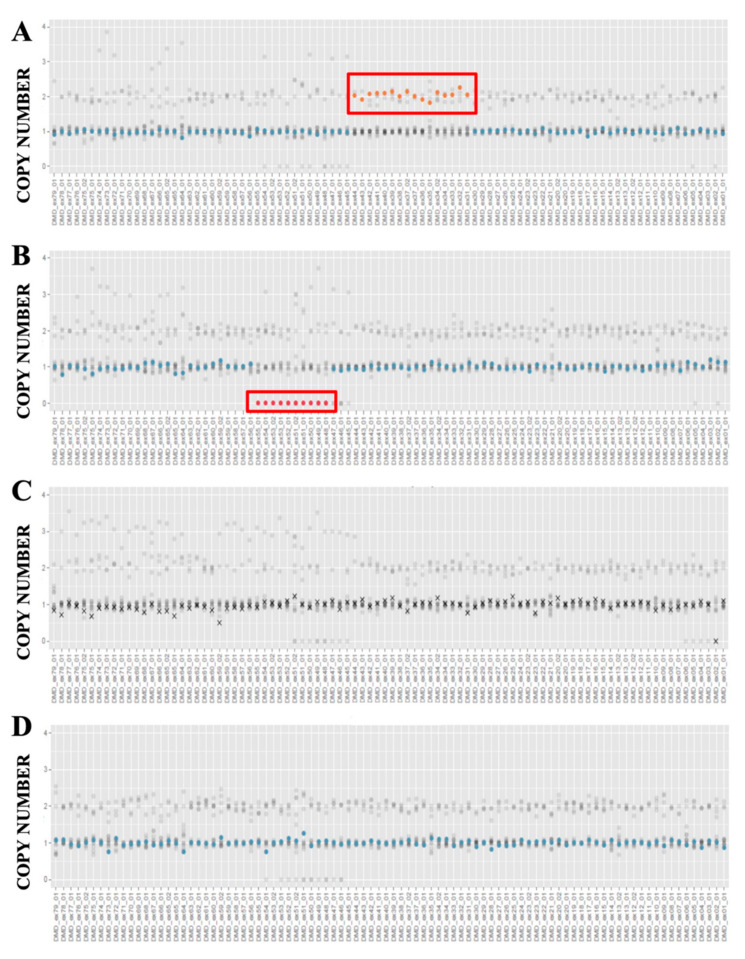
Examples of NGS-based CNV detection in *DMD* by Sophia Genetics Software. (**A**,**B**) Plots illustrate the presence of duplicated (**A**) or deleted (**B**) amplicons highlighted in red rectangles. (**C**) The panel displays a rejected sample for which despite the crosses along the entire gene, it is possible to notice the presence of a potential deletion at the end of the line (corresponding to the exon 2); (**D**) representation of the normal profile (blue dots indicate exons without CNVs) of a male with no GRs. The horizontal axis shows the exons (from exon 1 to the right, to exon 79 to the left) and the vertical axis the copy number value. CNV, copy number variation; GR, genomic rearrangement.

CNVs results in the remaining 34 patients were in alignment with the previous multiplex PCR and/or MLPA data. In detail, 11 samples presented duplications, 16 showed deletions and seven resulted wild type (Figure 3 and Figures S3–S5). In addition, Sophia Genetics software identified in two patients (ID-19 and ID-30) a single exon deleted/duplicated not confirmed using MLPA strategy; these can be considered as single dropouts (Table 2 and Figures S3–S5). 

### 3.4. Integrative Genome Viewer (IGV) Analysis

To make sure Sophia software did not miss any gene deletions, for each sample we performed a visual check of the reads corresponding to each *DMD* exon by IGV analysis. This control was carried out by comparing .bam file of samples with that of a reference male genome with no GRs in the *DMD* gene. In three patients “rejected” by Sophia DDM (ID-28, ID-43, ID-44 in Table 2), the IGV inspection allowed to reveal the deleted exons.

## 4. Discussion

Herein, the validation study of an NGS based-approach able to identify a unique strategy with both SNVs and GRs in the *DMD* gene is reported. 

The study group, including 45 unrelated patients, was previously screened using traditional approaches (multiplex PCR and/or MLPA for large deletions/duplications identification and Sanger sequencing for known point mutations) and re-analyzed in blind using NGS. All the male patients were affected by DMD, whereas the nine carrier females were asymptomatic. 

We found a total of 58 SNVs, including three nonsense pathogenic or likely pathogenic variants. One of them, the c.583C>T (p.Arg195*-rs398123999), was identified in a patient that resulted as a wild type for the previous traditional screening analysis not including the search for SNVs. Four patients were rejected from the CNVs analysis but recovered due to IGV software; for 36 individuals a CNV report was obtained. Five patients reported different undetermined regions, in particular, for the presence of duplications as specified in the Sophia Genetics CNV analysis report. All the variants found previously by using Sanger sequencing or MLPA were confirmed with our strategy. The molecular diagnosis of DMD/BMD has been traditionally considered as a lengthy and complex process [9,16]. Indeed, the procedure entailed both MLPA and/or multiplex PCR to detect large deletions/duplications and Sanger sequencing to detect SNVs, thus identifying all the possible *DMD* mutations [29]. However, considering the large size of the gene and the fact that SNVs occur more rarely than GRs, *DMD* Sanger analysis is not offered by all laboratories, with consequent lack of diagnostic sensitivity and underestimation of the relative weight that SNVs may have in DMD pathogenesis. Moreover, in most cases Sanger sequencing is performed only after that muscle biopsy reveals dystrophin deficiency, with consequent delays in obtaining a crucial information to identify the healthy carriers within the family, to offer to at-risk couples proper genetic counseling and the opportunity to take advantage of pre-implantation and/or prenatal diagnostic procedures [30,31,32]. In addition, the recent discovery of several emerging therapies, based on the repairing and/or restoring of specific mutations, requires the availability of even more accurate, sensitive, and specific molecular techniques for the fast and comprehensive *DMD* gene molecular scanning [16,33].

**Figure 4:**
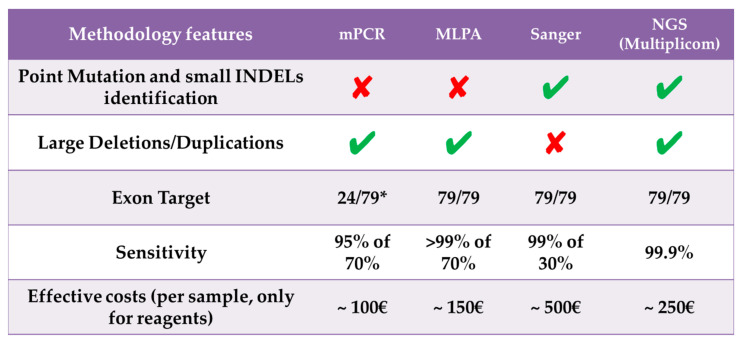
Comparison of the performances of traditional analytical approaches with respect to the NGS strategy used in the present study. *mPCR amplifies only mutational hotspots in 24 out of 79 exons of DMD gene. DMD, Duchenne muscular dystrophy; INDELs, small insertions and deletions; MLPA, multiplex ligation-dependent probe amplification; mPCR, multiplex polymerase chain reaction; NGS, next generation sequencing.

NGS offers an opportunity to fill-in existing gaps in the molecular diagnosis of DMD/BMD, as also recently proposed [34]. Thus, the method we described herein can be easily implemented in routine diagnostic practice with the advantage not only to reduce time and cost of the analysis but also to detect all the possible kinds of mutations affecting DMD (Figure 4). This increased sensitivity will, in turn, ameliorate the clinical care of affected patients and their families, and not least the number of muscle biopsies to be performed in the often very young patients.

Future perspectives concern the use of NGS as the first tier strategy followed by MLPA for negative patients, in order to exclude putative undetected duplications. To this end, we are expanding our case study by also enrolling undiagnosed patients. NGS drawbacks may be indicated as follows: (i) the methodology does not analyze deep intronic regions; (ii) the cost–benefit assessment, which depends on the number of samples tested for each sequencing run, should be calculated by each laboratory depending on patient influx and urgency issues.

## Figures and Tables

**Table 1:** *DMD* small nucleotide variants identified by NGS in the 40 analyzed subjects. Fifty-eight variants were identified in total, of which five pathogenic mutations (in bold) were identified in one study subject, as reported in Table 2.

HGVS * Coding (cDNA)	HGVS * Protein Level	Reference SNP ID Number (rs)	Clinical Significance (ClinVar)	ACMG Classification **	DANN Score ^§^	MAF ^#^
c.94-9dupT		rs3834997	Benign/likely benign	Benign	NA	0.0923
c.530+7A>T		rs72470523	Likely Benign	Likely Benign	0.63	0.00007
**c.583C>T**	p.Arg195 *	**rs398123999**	**Pathogenic**	**Pathogenic**	**0.99**	**NA**
c.733A>G	p.Ile245Val	rs140510985	UCV	UCV	0.98	0.00004
c.832A>G	p.Ile278Val	rs779964937	NR.	UCV	0.99	0.0000135
c.1483-67A>T		rs1435727	Likely Benign	Benign	0.29	0.039
c.1483-72T>C		rs17309542	NR	Benign		0.0698
c.1483-110G>A		rs808543	Benign	Benign	0.47	0.518
c.1635A>G	p.Arg545=	rs5927083	Benign/likely benign	Benign	0.54	0.107
c.1704+51T>C		rs5927082	Benign	Benign	0.33	0.0968
c.1812C>T	p.Ala604=	rs140919039	Benign/likely benign	Benign	0.19	0.000268
c.1993-37T>G		rs115571	Benign	Benign	0.75	0.724
c.2168+13T>C		rs228373	Benign/likely benign	Benign	0.77	0.313
**c.2414C>G**	p.Ser805 *	**NR**	**NR**	**Pathogenic**	**0.99**	**NA**
c.2645A>G	p.Asp882Gly	rs228406	Benign	Benign	0.80	0.728
c.2827C>T	p.Arg943Cys	rs199986217	Benign/likely benign	Benign	0.99	0.000191
**c.3259C>T**	p.Gln1087 *	**rs886039536**	**Pathogenic**	**Pathogenic**	**0.99**	**NA**
c.3603+15dupA		rs5902031	Likely benign	UCV	NA	0.428
c.3603+15delA		rs745638361	Benign	UCV	NA	0.0902
c.3787-18T>C		rs72468656	Benign	Benign	0.76	0.00633
**c.4120delG**	p.Glu1374Argfs*8	**NR**	**NR**	**Pathogenic**	**NA**	**NA**
c.4234-13A>G		rs41303181	Benign/likely benign	Benign	0.31	0.0607
c.4519-34T>A		rs72468639	Benign	Benign	0.56	0.0196
c.4675-53G>T		rs72468636	Likely benign	Benign	0.26	0.0115
c.5155-63T>A		rs12387861	NR	Benign	0.57	0.0346
c.5234G>A	p.Arg1745His	rs1801187	Benign/likely benign	Benign	0.99	0.528
c.5326-54A>C		rs41309607	Likely benign	Benign	0.83	0.00777
c.5586+94_5586+95dupCT		rs5902025	NR	Benign	NA	0.767
**c.5697dupA**	p.Leu1900Ilefs*6	**rs794727661**	**Pathogenic**	**Pathogenic**	**NA**	**NA**
c.5739+15G>T		rs398123996	NR	UCV	0.77	0.0000164
c.5740-67G>T		rs6527184	NR	Benign	0.79	0.169
c.5922+11A>C		rs1394206261	Likely benign	Likely benign	0.78	0.00000561
c.6118-63_6118-62dupAT		rs3842480	NR	Benign	NA	0.25
c.6290+27T>A		rs3788896	NR	Benign	0.45	0.119
c.6291-115G>A		rs3747400	Benign	Benign	0.28	0.273
c.6463C>T	p.Arg2155Trp	rs1800273	Benign/likely benign	Benign	0.99	0.0261
c.6614+26G>T		rs3761604	Benign	Benign	0.49	0.329
HGVS * Coding (cDNA)	HGVS * **Protein Level**	**Reference SNP ID Number (rs)**	**Clinical Significance (ClinVar)**	**ACMG** Classification **	**DANN Score ^§^**	**MAF ^#^**
c.7096A>C	p.Gln2366Lys	rs1800275	Benign	Benign	0.98	0.234
c.7310-36C>T		rs72466586	Benign	Benign	0.83	0.011
c.7542+13A>G		rs72466585	Benign	Benign	0.61	0.00384
c.7728T>C	p.Asn2576=	rs1801188	Benign/likely benign	Benign	0.66	0.17
c.8027+11C>T		rs2270672	Benign/likely benign	Benign	0.67	0.332
c.8669-75C>G		rs17338583	Benign	Benign	0.53	0.0723
c.8729A>T	p.Glu2910Val	rs41305353	Benign/likely benign	Benign	0.99	0.0209
c.8734A>G	p.Asn2912Asp	rs1800278	Benign/likely benign	Benign	0.98	0.0213
c.8762A>G	p.His2921Arg	rs1800279	Benign/likely benign	Benign	0.69	0.0256
c.8810G>A	p.Gln2937Arg	rs1800280	Benign	Benign	0.83	0.898
c.9164-145A>G		rs7059188	Benign	Benign	0.82	0.071
c.9563+118C>A		NR	NR	UCV	0.46	NA
c.9564-97C>T		rs2293667	Benign	Benign	0.42	0.843
c.9649+15T>C		rs2293668	Benign/likely benign	Benign	0.75	0.867
c.9808-63G>A		NR	NR	UCV	0.43	NA
c.9974+22dupA		rs3833412	NR	Benign	NA	0.411
c.9974+22delA		rs3833412	NR	Benign	NA	0.114
c.10087-20C>T		rs41303187	Benign/likely benign	Benign	0.76	0.00194
c.10328+67G>A		rs2404496	NR	Benign	0.59	0.872
c.10797+42C>G		rs72466537	NR	Benign	0.68	0.00731
c.10554-36_10554-33del		rs200534475	NR	UCV	NA	0.746

* Variants nomenclature according to Human Genome Variation Society (HGVS) guidelines; ** ACMG: American College of Medical Genetics; ^§^ DANN: Deleterious Annotation of genetic variants using Neural Networks; ^#^ MAF: Minor allele frequency, based on the Genome Aggregation database (gnomeAD); DANN, deleterious annotation of genetic variants using neural networks; NA, not available; NR, not reported; UCV: Uncertain significance variant. In bold are highlighted all the pathogenic variants found.

**Table 2:** Comparison of the results found by combining MLPA and multiplex-PCR, or by Sanger sequencing approaches (“Previous results”), and those obtained by next generation sequencing (“NGS results), in the 40 subjects enrolled in this study. For the CNV analysis four samples were “REJECTED” and five samples showed “UNDETERMINED” regions, due to a high background noise level. In the NGS point mutation’s results column, a pathogenic mutation not previously identified by the traditional diagnostic flowchart is highlighted in bold.

			Previous Results			NGS Results	
Sample ID	Gender	Phenotype	mPCR Results	MLPA Results	Sanger Point Mutation Results	NGS CNV Results	NGS Point Mutation Results *
**1**	M	Patient	WT	Ex13-29 dup	n.p.	Ex13-29 dup	c.8762A>G p.His2921Arg
**2**	M	Patient	WT	Ex57-65 dup	n.p.	Ex57-65 dup	-
**3**	M	Patient	WT	Ex10-11 dup	n.p.	UNDETERMINED Ex10	-
**4**	F	Carrier	WT	Ex44-59;64-79 dup	n.p.	Ex44-59;64-79 dup	c.7310-36C>T (het); c.9808-63G>A (het)
**5**	M	Patient	WT	WT	n.p.	WT	c.583C>T (p.Arg195 *)
**8**	M	Patient	WT	Ex54 dup	n.p.	UNDETERMINED Ex54	-
**9**	M	Patient	WT	Ex54dup	n.p.	UNDETERMINED Ex54	-
**10**	M	Patient	WT	Ex2 dup	n.p.	UNDETERMINED Ex2	-
**11**	M	Patient	WT	Ex55 del	n.p.	Ex55 del	-
**12**	F	Carrier	n.p.	n.p.	c.2414C>G (p.Ser805*)	WT	c.2414C>G (p.Ser805*); c.4519-34T>A (het); c.4675-53G>T (het); c.5155-63T>A (het)
**13**	M	Patient	WT	WT	c.5697dupA (p.Leu1900Ilefs*6)	WT	c.5697dupA (p.Leu1900Ilefs*6)
**14**	M	Patient	n.p.	n.p.	c.4120delG (p.Glu1374Argfs*8)	WT	c.4120delG (p.Glu1374Argfs*8); c.9563+118C>A
**15**	F	Carrier	Ex44 del	n.p.	n.p.	Ex44 del	c.5326-54A>C (het); c.8729A>T (p.Glu2910Val) (het); c.8734A>G (p.Asn2912Asp) (het); c.10797+42C>G (het)
**16**	M	Patient	Ex13;17;19 dup	Ex13-29 dup	n.p.	Ex13-29 dup	c.8762A>G (p.His2921Arg)
**17**	M	Patient	Ex48 del	Ex48 del	n.p.	Ex48 del	c.8762A>G (p.His2921Arg)
**18**	M	Patient	Ex41-44 dup	Ex31-44 dup	n.p.	Ex31-44 dup	c.3787-18T>C; c.8762A>G (p.His2921Arg)
**19**	M	Patient	Ex2 dup	Ex2 dup	n.p.	UNDETERMINED Ex2;75	c.733A>G (p.Ile245Val)
**20**	M	Patient	Ex45-47;50-53 dup	Ex45-47;50-62 dup	n.p.	Ex45-47;50-62 dup	c.5326-54A>C
**22**	M	Patient	Ex12;13;17 dup	Ex10-17 dup	n.p.	Ex10-17 dup	c.1483-67A>T
**23**	F	Carrier	n.p.	n.p.	c.3259C>T (p.Gln1087*)	WT	c.3259C>T (p.Gln1087*) (het); c.832A>G (p.Ile278Val) (het)
**24**	M	Patient	Ex48-53 del	Ex48-55 del	n.p.	Ex48-55 del	-
**25**	M	Patient	Ex45-53 del	Ex45-55 del	n.p.	Ex45-55 del	-
**26**	M	Patient	Ex45-52 del	n.p.	n.p.	Ex45-52 del	-
**28**	M	Patient	Ex45-47del	n.p.	n.p.	REJECTED	c.7310-36C>T
**29**	F	Carrier	Ex45-48 del	Ex45-48 del	n.p.	Ex45-48 del	c.6463C>T (p.Arg2155Trp) (homo); c.7542+13A>G (het); c.8762A>G (p.His2921Arg) (het)
**30**	F	Carrier	n.p.	Ex26-30 del	n.p.	Ex13;26-30 del	c.1483-67A>T (het); c.4519-34T>A (het); c.5155-63T>A (het)
**31**	F	Carrier	Ex45-52 del	n.p.	n.p.	Ex45-52 del	-
**32**	F	Carrier	Ex46-48 del	n.p.	n.p.	Ex46-48 del	-
**34**	M	Patient	Ex17;19 dup	Ex14-20 dup	n.p.	Ex14-20 dup	-
**35**	F	Carrier	Ex12-19 del	Ex10-29 del	n.p.	Ex10-29 del	-
**38**	M	Patient	n.p.	Ex48 del	n.p.	Ex48 del	c.2827C>T (p.Arg943Cys); c.3787-18T>C; c.8762A>G (p.His2921Arg)
**40**	M	Patient	Ex45-52 del	Ex45-55 del	n.p.	Ex45-55 del	-
**41**	M	Patient	Ex45-47 del	Ex45-47 del	n.p.	Ex45-47 del	c.530+7A>T; c.1812C>T (p.Ala604=)
**42**	M	Patient	Ex48-51 del	Ex48-51 del	n.p.	Ex48-51 del	c.10087-20C>T
**43**	M	Patient	Ex2 del	Ex2 del	n.p.	REJECTED	-
**44**	M	Patient	Ex2-6 del	Ex2-7 del	n.p.	REJECTED	-
**45**	M	Patient	Ex46-51 del	Ex46-51 del	n.p.	Ex46-51 del	c.4519-34T>A; c.4675-53G>T; c.5155-63T>A; c.10087-20C>T
**46**	M	Patient	n.p.	n.p.	n.p.	WT	c.4519-34T>A; c.4675-53G>T; c.5155-63T>A
**47**	M	Patient	n.p.	n.p.	n.p.	WT	-
**48**	M	Patient	n.p.	n.p.	n.p.	REJECTED	c.5326-54A>C

* In the table are reported only the variants with minor allele frequency (MAF) ≤0.05. CNV, copy number variation; M: Male; F: Female; Ex: Exon; del: Deletion; dup: Duplication; n.p.: not performed; WT: wild type; mPCR, multiplex PCR; het, heterozygous; homo, homozygous. In bold the new pathogenic variant found using NGS strategy.

## Supplementary Materials

The following are available online at https://www.mdpi.com/article/10.3390/diagnostics11101910/s1, Table S1: Percentage of target regions at different values of sequencing coverage, Table S2: General run statistics of the CNV analysis; Figure S1: Panel of rejected samples for CNV analysis, Figure S2: Panel of samples with undetermined regions, Figure S3: NGS-based deletion detection in DMD by Sophia Genetics Software, Figure S4: NGS-based duplication detection in DMD by Sophia Genetics Software, Figure S5: Panel of wild-type samples for CNV analysis.

## References

[1] Duan D., Goemans N., Takeda S., Mercuri E., Aartsma-Rus A. (2021). Duchenne muscular dystrophy. Nat. Rev. Dis. Prim..

[2] Waldrop M.A., Flanigan K.M. (2019). Update in Duchenne and Becker muscular dystrophy. Curr. Opin. Neurol..

[3] Esposito G., Carsana A. (2019). Metabolic Alterations in Cardiomyocytes of Patients with Duchenne and Becker Muscular Dystrophies. J. Clin. Med..

[4] Narayanan K., Bougouin W., Sharifzadehgan A., Waldmann V., Karam N., Marijon E., Jouven X. (2017). Sudden Cardiac Death During Sports Activities in the General Population. Card. Electrophysiol. Clin..

[5] Paolella G., Pisano P., Albano R., Cannaviello L., Mauro C., Esposito G., Vajro P. (2012). Fatty liver disease and hypertransaminasemia hiding the association of clinically silent Duchenne muscular dystrophy and hereditary fructose intolerance. Ital. J. Pediatr..

[6] Falzarano M.S., Scotton C., Passarelli C., Ferlini A. (2015). Duchenne muscular dystrophy: From diagnosis to therapy. Molecules.

[7] Wein N., Alfano L., Flanigan K.M. (2015). Genetics and Emerging Treatments for Duchenne and Becker Muscular Dystrophy. Pediatr. Clin. N. Am..

[8] Le Rumeur E., Rumeur E. (2015). Le Dystrophin and the two related genetic diseases, Duchenne and Becker muscular dystrophies. Bosn. J. Basic Med. Sci..

[9] Aartsma-Rus A., Ginjaar I.B., Bushby K. (2016). The importance of genetic diagnosis for Duchenne muscular dystrophy. J. Med. Genet..

[10] Esposito G., Tremolaterra M.R., Marsocci E., Tandurella I.C., Fioretti T., Savarese M., Carsana A. (2017). Precise mapping of 17 deletion breakpoints within the central hotspot deletion region (introns 50 and 51) of the DMD gene. J. Hum. Genet..

[11] Crone M., Mah J.K. (2018). Current and Emerging Therapies for Duchenne Muscular Dystrophy. Curr Treat. Options Neurol..

[12] Nallamilli B.R.R., Ankala A., Hegde M. (2014). Molecular diagnosis of duchenne muscular dystrophy. Curr. Protoc. Hum. Genet..

[13] Fratter C., Dalgleish R., Allen S.K., Santos R., Abbs S., Tuffery-Giraud S., Ferlini A. (2020). EMQN best practice guidelines for genetic testing in dystrophinopathies. Eur. J. Hum. Genet..

[14] Esposito G., Ruggiero R., Savarese M., Savarese G., Tremolaterra M.R., Salvatore F., Carsana A. (2013). Prenatal molecular diagnosis of inherited neuromuscular diseases: Duchenne/Becker muscular dystrophy, myotonic dystrophy type 1 and spinal muscular atrophy. Clin. Chem. Lab. Med..

[15] Kumar K.R., Cowley M.J., Davis R.L. (2019). Next-Generation Sequencing and Emerging Technologies. Semin. Thromb. Hemost..

[16] Bello L., Pegoraro E. (2016). Genetic diagnosis as a tool for personalized treatment of Duchenne muscular dystrophy. Acta Myol. Myopathies Cardiomyopathies Off. J. Mediterr. Soc. Myol..

[17] Levy S.E., Myers R.M. (2016). Advancements in Next-Generation Sequencing. Annu. Rev. Genomics Hum. Genet..

[18] Limongelli G., Nunziato M., D’Argenio V., Esposito M.V., Monda E., Mazzaccara C., Caiazza M., D’Aponte A., D’Andrea A., Bossone E. (2020). Yield and clinical significance of genetic screening in elite and amateur athletes. Eur. J. Prev. Cardiol..

[19] McInerney-Leo A.M., Duncan E.L. (2021). Massively Parallel Sequencing for Rare Genetic Disorders: Potential and Pitfalls. Front. Endocrinol..

[20] Cock-Rada A.M., Ossa C.A., Garcia H.I., Gomez L.R. (2018). A multi-gene panel study in hereditary breast and ovarian cancer in Colombia. Fam. Cancer.

[21] Nunziato M., Esposito M.V., Starnone F., Diroma M.A., Calabrese A., del Monaco V., Buono P., Frasci G., Botti G., D’Aiuto M. (2018). A multi-gene panel beyond BRCA1/BRCA2 to identify new breast cancer-predisposing mutations by a picodroplet PCR followed by a next-generation sequencing strategy: A pilot study. Anal. Chim. Acta.

[22] Nerakh G., Ranganath P., Murugan S. (2021). Next-Generation Sequencing in a Cohort of Asian Indian Patients with the Duchenne Muscular Dystrophy Phenotype: Diagnostic Yield and Mutation Spectrum. J. Pediatr. Genet..

[23] Fioretti T., Auricchio L., Piccirillo A., Vitiello G., Ambrosio A., Cattaneo F., Ammendola R., Esposito G. (2020). Multi-gene next-generation sequencing for molecular diagnosis of autosomal recessive congenital ichthyosis: A genotype-phenotype study of four Italian patients. Diagnostics.

[24] Liu X., Tao T., Zhao L., Li G., Yang L. (2021). Molecular diagnosis based on comprehensive genetic testing in 800 Chinese families with non-syndromic inherited retinal dystrophies. Clin. Exp. Ophthalmol..

[25] Yao R., Yu T., Qing Y., Wang J., Shen Y. (2019). Evaluation of copy number variant detection from panel-based next-generation sequencing data. Mol. Genet. Genom. Med..

[26] Nallamilli B.R.R., Chaubey A., Valencia C.A., Stansberry L., Behlmann A.M., Ma Z., Mathur A., Shenoy S., Ganapathy V., Jagannathan L. (2021). A single NGS-based assay covering the entire genomic sequence of the DMD gene facilitates diagnostic and newborn screening confirmatory testing. Hum. Mutat..

[27] Nunziato M., Starnone F., Lombardo B., Pensabene M., Condello C., Verdesca F., Carlomagno C., De Placido S., Pastore L., Salvatore F. (2017). Fast detection of a BRCA2 large genomic duplication by next generation sequencing as a single procedure: A case report. Int. J. Mol. Sci..

[28] Carsana A., Frisso G., Intrieri M., Tremolaterra M.R., Savarese G., Scapagnini G., Esposito G., Santoro L., Salvatore F. (2010). A 15-year molecular analysis of DMD/BMD: Genetic features in a large cohort. Front. Biosci.-Elit..

[29] Okubo M., Minami N., Goto K., Goto Y., Noguchi S., Mitsuhashi S., Nishino I. (2016). Genetic diagnosis of Duchenne/Becker muscular dystrophy using next-generation sequencing: Validation analysis of DMD mutations. J. Hum. Genet..

[30] Dinh L.T., Tran V.K., Luong L.H., Le P.T., Nguyen A.D., Thi Nguyen B.S., Chi D.V., Tran T.H., Bui T.H., Van Ta T. (2019). Assessment of 6 STR loci for prenatal diagnosis of Duchenne Muscular Dystrophy. Taiwan J. Obstet. Gynecol..

[31] Zhang J., Ma D., Liu G., Wang Y., Liu A., Li L., Luo C., Hu P., Xu Z. (2019). Genetic analysis of 62 Chinese families with Duchenne muscular dystrophy and strategies of prenatal diagnosis in a single center. BMC Med. Genet..

[32] Ren Y., Lian Y., Yan Z., Zhai F., Yang M., Zhu X., Wang Y., Nie Y., Guan S., Kuo Y. (2021). Clinical application of an NGS-based method in the preimplantation genetic testing for Duchenne muscular dystrophy. J. Assist. Reprod. Genet..

[33] Ebrahimzadeh-Vesal R., Teymoori A., Aziminezhad M., Hosseini F.S. (2018). Next Generation Sequencing approach to molecular diagnosis of Duchenne muscular dystrophy; identification of a novel mutation. Gene.

[34] Kong X., Zhong X., Liu L., Cui S., Yang Y., Kong L. (2019). Genetic analysis of 1051 Chinese families with Duchenne/Becker Muscular Dystrophy. BMC Med. Genet..

