# Supplementary material for: Comprehensive Molecular Analysis of DMD Gene Increases the Diagnostic Value of Dystrophinopathies: A Pilot Study in a Southern Italy Cohort of Patients
Source: Diagnostics (Basel). 2021 Oct 15;11(10):1910. doi: 10.3390/diagnostics11101910 (PMC8534830; doi:10.3390/diagnostics11101910)
Supplement: Supplementary file 1 [file diagnostics-11-01910-s001.zip › diagnostics-1347585-supplementary.pdf]

Supplementary Materials

Comprehensive Molecular Analysis of *DMD* Gene Increases the Diagnostic Value of Dystrophinopathies: A Pilot Study in a Southern Italy Cohort of Patients

Fatima Domenica Elisa De Palma [1,2,†], Marcella Nunziato [1,2,†], Valeria D'Argenio [1,3], Maria Savarese [1,2], Gabriella Esposito [1,2,*] and Francesco Salvatore [1,2,*]

**Content**	
Table S1	Pag. 2
Table S2	Pag. 3
Figure S1	Pag. 4
Figure S2	Pag. 5
Figure S3	Pag. 6
Figure S4	Pag. 7
Figure S5	Pag. 8

Table S1. Percentage of target regions at different values of sequencing coverage.

N°	Sample ID	20×	30×	50×	100×	500×	1000×
1	**1**	100.00%	100.00%	99.99%	99.99%	74.67%	46.47%
2	**2**	100.00%	100.00%	100.00%	99.99%	95.72%	67.51%
3	**3**	100.00%	100.00%	99.99%	99.99%	89.36%	57.32%
4	**4**	100.00%	100.00%	100.00%	100.00%	96.74%	73.86%
5	**5**	100.00%	100.00%	100.00%	95.74%	73.04%	67.95%
6	**8**	100.00%	100.00%	100.00%	99.99%	81.75%	59.47%
7	**9**	100.00%	100.00%	100.00%	99.99%	79.09%	63.52%
8	**10**	100.00%	100.00%	100.00%	100.00%	91.07%	68.80%
9	**11**	99.00%	99.00%	99.00%	98.99%	80.49%	70.53%
10	**12**	100.00%	100.00%	100.00%	100.00%	82.56%	69.78%
11	**13**	100.00%	100.00%	99.99%	99.99%	79.38%	61.70%
12	**14**	100.00%	100.00%	100.00%	99.99%	87.23%	68.17%
13	**15**	100.00%	100.00%	99.99%	99.99%	91.58%	59.69%
14	**16**	100.00%	100.00%	99.99%	99.99%	81.72%	56.27%
15	**17**	99.07%	99.07%	99.07%	99.06%	89.10%	61.57%
16	**18**	100.00%	100.00%	99.99%	99.99%	83.94%	62.29%
17	**19**	100.00%	100.00%	99.99%	99.99%	90.59%	57.35%
18	**20**	100.00%	100.00%	99.99%	99.99%	80.92%	63.42%
19	**22**	100.00%	100.00%	99.99%	99.99%	89.53%	66.89%
20	**23**	100.00%	100.00%	100.00%	100.00%	96.76%	75.00%
21	**24**	91.68%	91.68%	91.68%	91.67%	86.40%	65.32%
22	**25**	89.08%	89.08%	89.08%	88.17%	65.97%	62.67%
23	**26**	92.52%	92.52%	90.28%	77.07%	66.95%	63.02%
24	**28**	97.40%	97.40%	97.39%	97.39%	95.68%	85.35%
25	**29**	100.00%	100.00%	100.00%	100.00%	94.28%	70.12%
26	**30**	100.00%	100.00%	100.00%	99.99%	78.60%	64.59%
27	**31**	100.00%	100.00%	100.00%	99.99%	96.08%	77.74%
28	**32**	100.00%	100.00%	100.00%	100.00%	96.18%	79.53%
29	**34**	100.00%	100.00%	100.00%	100.00%	95.38%	71.33%
30	**35**	100.00%	100.00%	100.00%	100.00%	88.96%	51.13%
31	**38**	99.07%	99.07%	99.07%	99.06%	96.39%	78.13%
32	**40**	89.08%	89.08%	89.08%	89.08%	89.07%	79.49%
33	**41**	97.40%	97.40%	97.39%	97.39%	90.26%	46.01%
34	**42**	95.89%	95.89%	95.89%	95.88%	89.64%	50.97%
35	**43**	99.19%	99.19%	99.18%	99.18%	93.87%	61.71%
36	**44**	68.85%	68.85%	68.85%	68.83%	66.91%	51.71%
37	**45**	94.15%	94.15%	94.14%	94.14%	93.12%	81.99%
38	**46**	100.00%	100.00%	100.00%	99.99%	98.64%	92.74%
39	**47**	100.00%	100.00%	100.00%	99.99%	99.99%	86.57%
40	**48**	100.00%	100.00%	99.99%	99.99%	98.64%	90.07%

Table S2. General run statistics of the CNV analysis.

N°	Sample ID	Average Coverage per Region	Min. Average Coverage per Plex	Residual Noise	Noise Status
1	**1**	1150	334	0.081	low-noise
2	**2**	1850	728	0.08	medium-noise
3	**3**	1306	797	0.065	low-noise
4	**4**	1708	770	0.062	low-noise
5	**5**	2042	216	0.079	medium-noise
6	**8**	1844	529	0.078	medium-noise
7	**9**	1890	457	0.064	low-noise
8	**10**	1891	623	0.067	low-noise
9	**11**	1894	501	0.08	medium-noise
10	**12**	1666	519	0.07	low-noise
11	**13**	1440	481	0.062	low-noise
12	**14**	1771	575	0.063	low-noise
13	**15**	1370	678	0.061	low-noise
14	**16**	1669	454	0.062	low-noise
15	**17**	1655	686	0.068	low-noise
16	**18**	1577	519	0.053	low-noise
17	**19**	1532	715	0.064	low-noise
18	**20**	1544	441	0.069	low-noise
19	**22**	1549	796	0.075	low-noise
20	**23**	1782	1016	0.066	low-noise
21	**24**	1532	754	0.084	low-noise
22	**25**	1550	270	0.101	medium-noise
23	**26**	1587	101	0.1	medium-noise
24	**28**	2154	1229	0.132	rejected
25	**29**	1439	1053	0.077	low-noise
26	**30**	1793	429	0.08	medium-noise
27	**31**	1561	1392	0.069	low-noise
28	**32**	1514	1366	0.07	medium-noise
29	**34**	1422	1100	0.068	low-noise
30	**35**	1171	999	0.069	low-noise
31	**38**	1640	1113	0.078	low-noise
32	**40**	1869	1729	0.065	low-noise
33	**41**	1119	875	0.095	medium-noise
34	**42**	1120	941	0.092	medium-noise
35	**43**	1343	1112	0.126	rejected
36	**44**	1108	1	0.533	rejected
37	**45**	1795	1273	0.072	low-noise
38	**46**	2367	1541	0.103	medium-noise
39	**47**	1874	1396	0.099	medium-noise
40	**48**	1822	1473	0.17	rejected

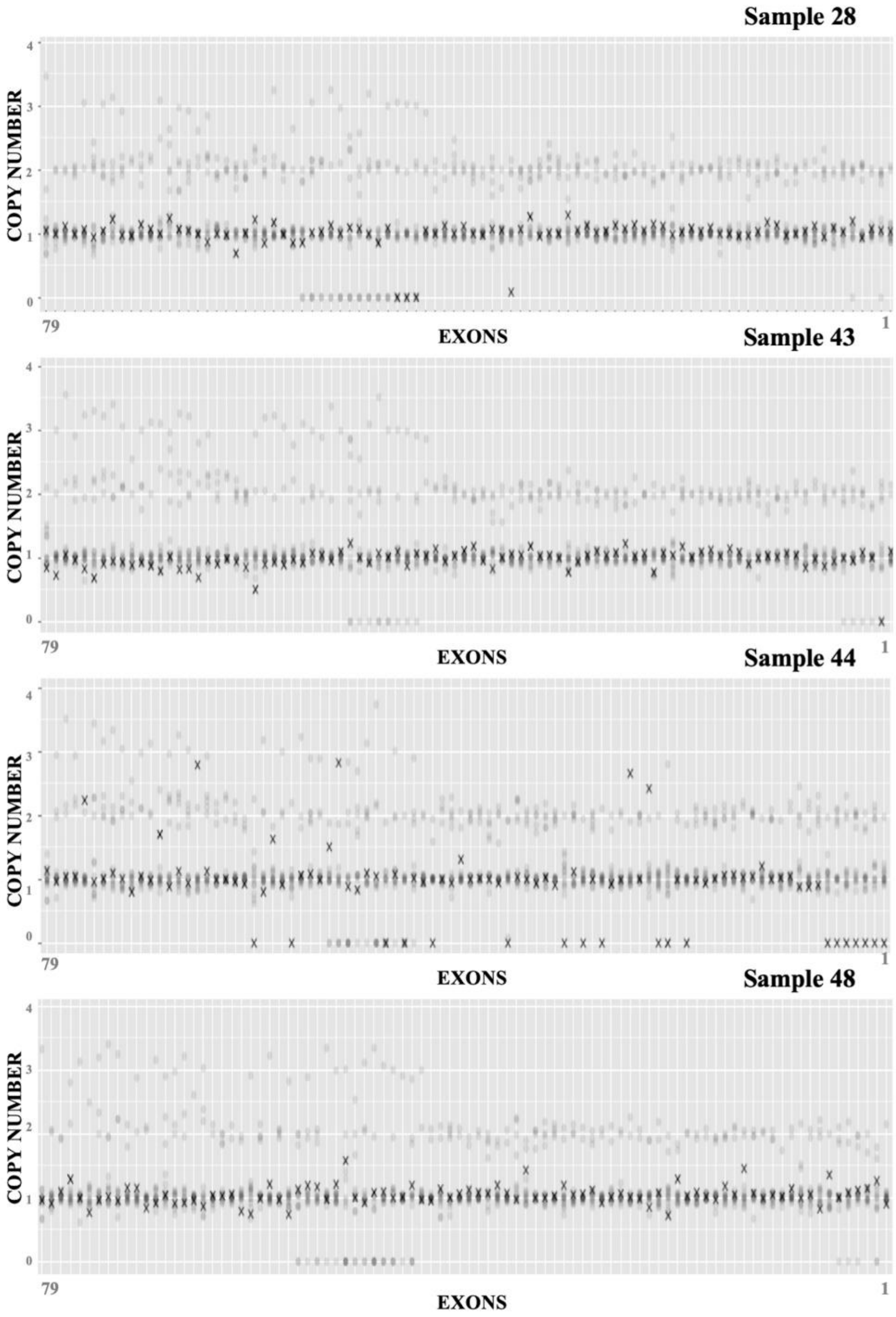

Figure S1. Panel of rejected samples for CNV analysis. Four samples were rejected automatically by the software Sophia Genetics for CNV analysis. The horizontal axis shows the exons and the vertical axis the copy number value.

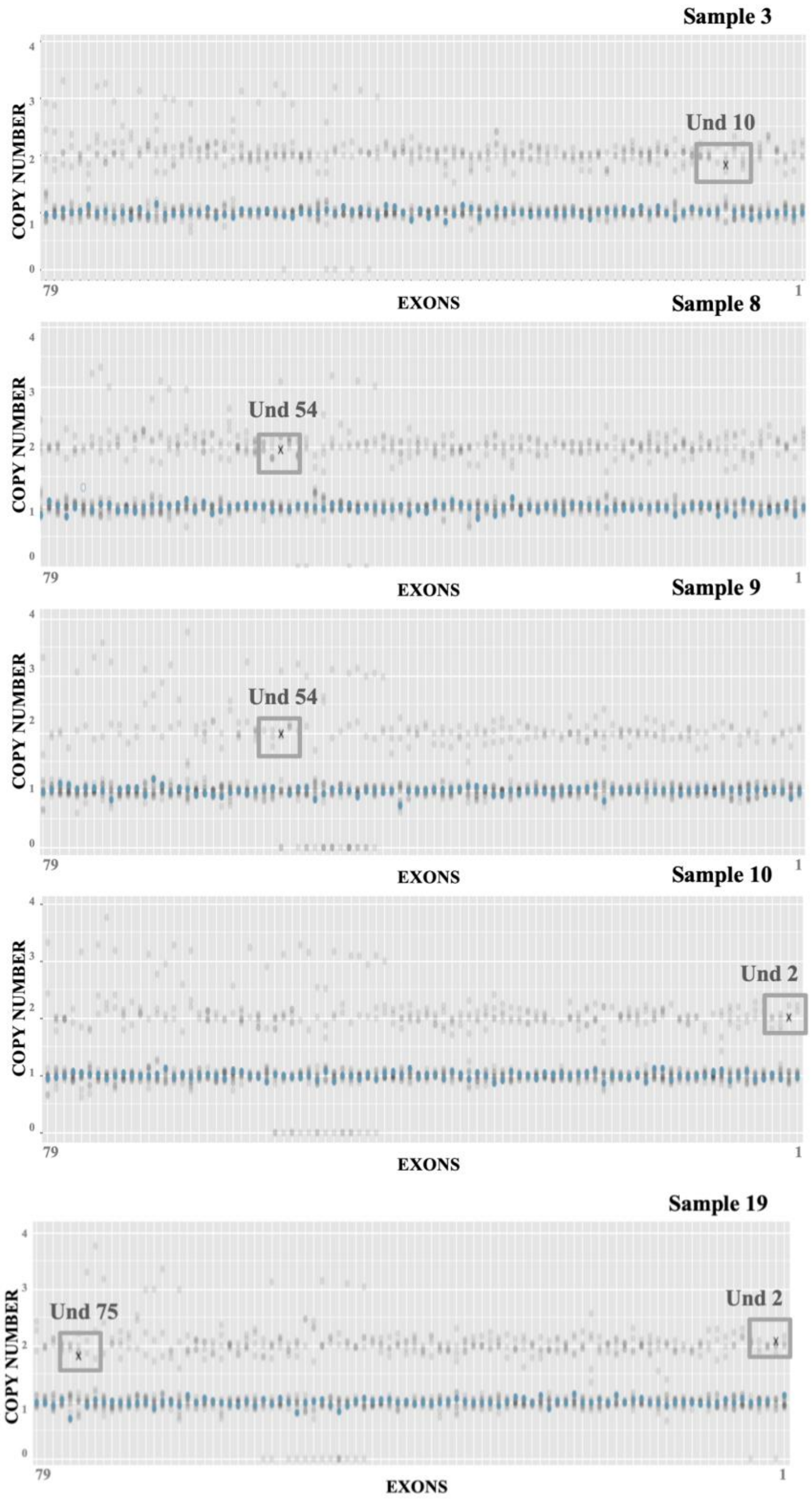

Figure S2. Panel of samples with undetermined regions. The CNV analysis performed by Sophia Genetics software shows 5 samples with some undetermined regions identified by a cross and marked in the figure (Und: Undetermined).

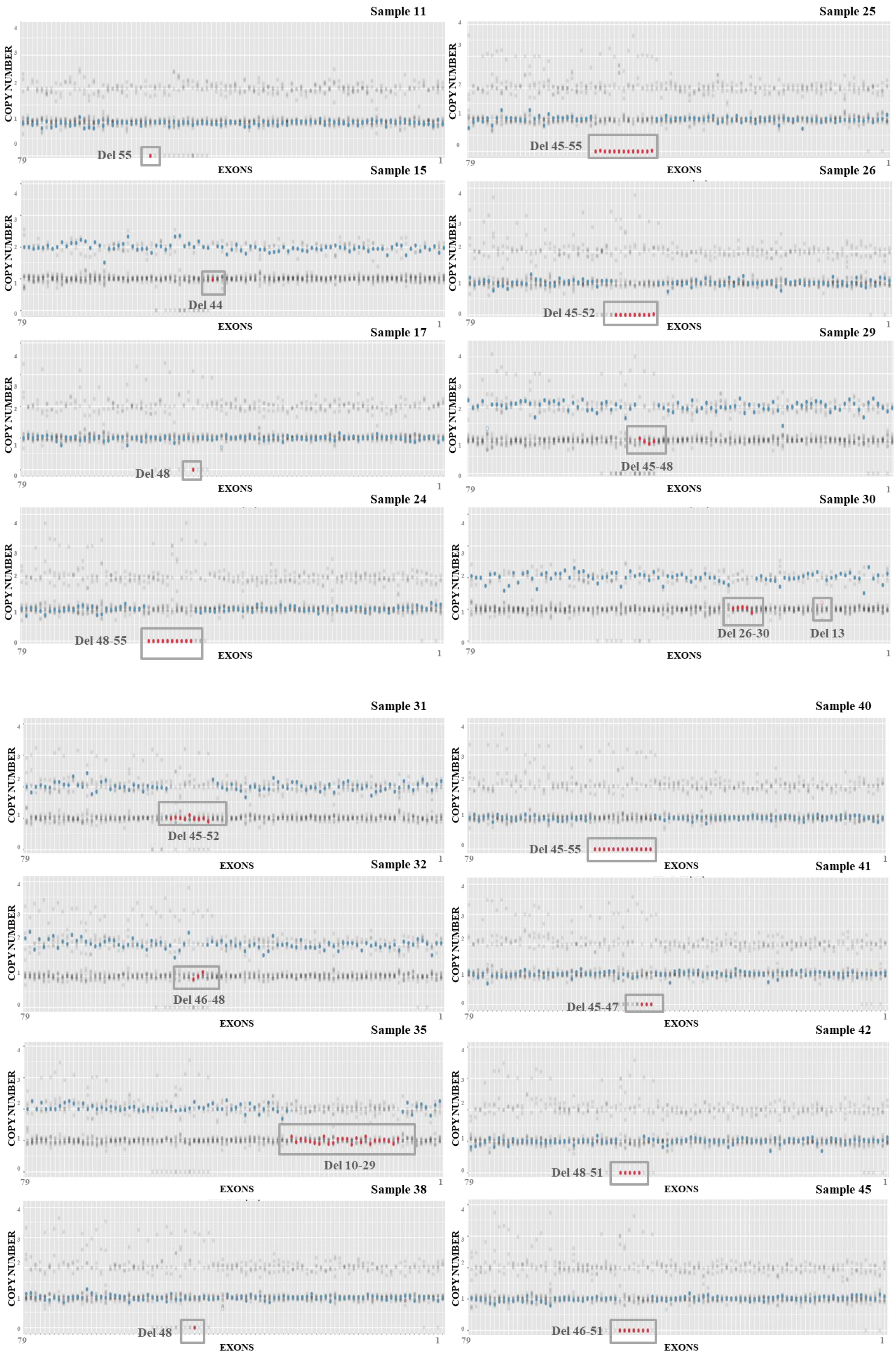

Figure S3. NGS-based deletion detection (red color) in DMD by Sophia Genetics Software. Sixteen samples were identi-fied as deleted after the bioinformatic analysis of the NGS data.

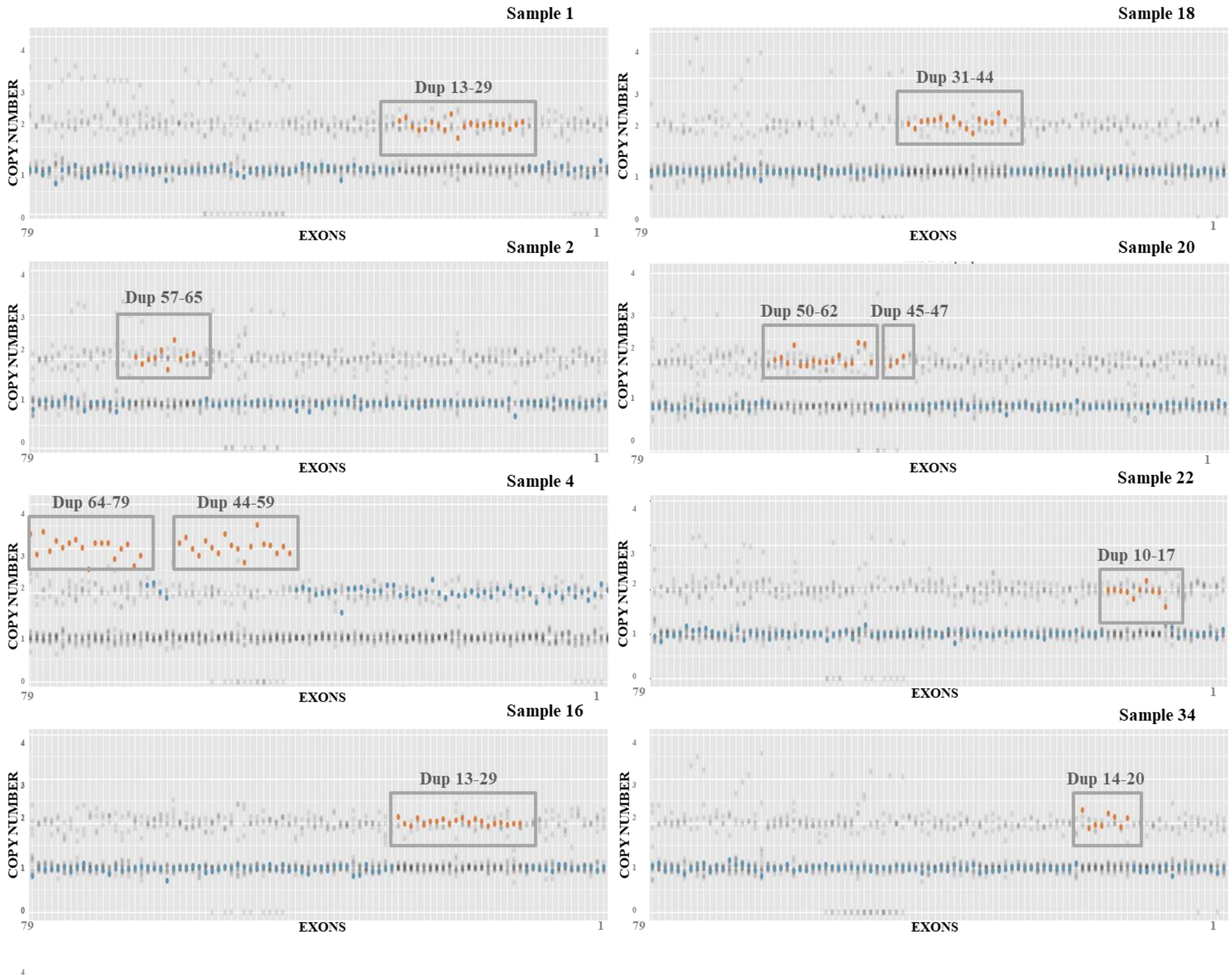

Figure S4. NGS-based duplication detection (orange color) in DMD by Sophia Genetics Software. Eight samples were detected as duplicated by the bioinformatic evaluation of the NGS data.

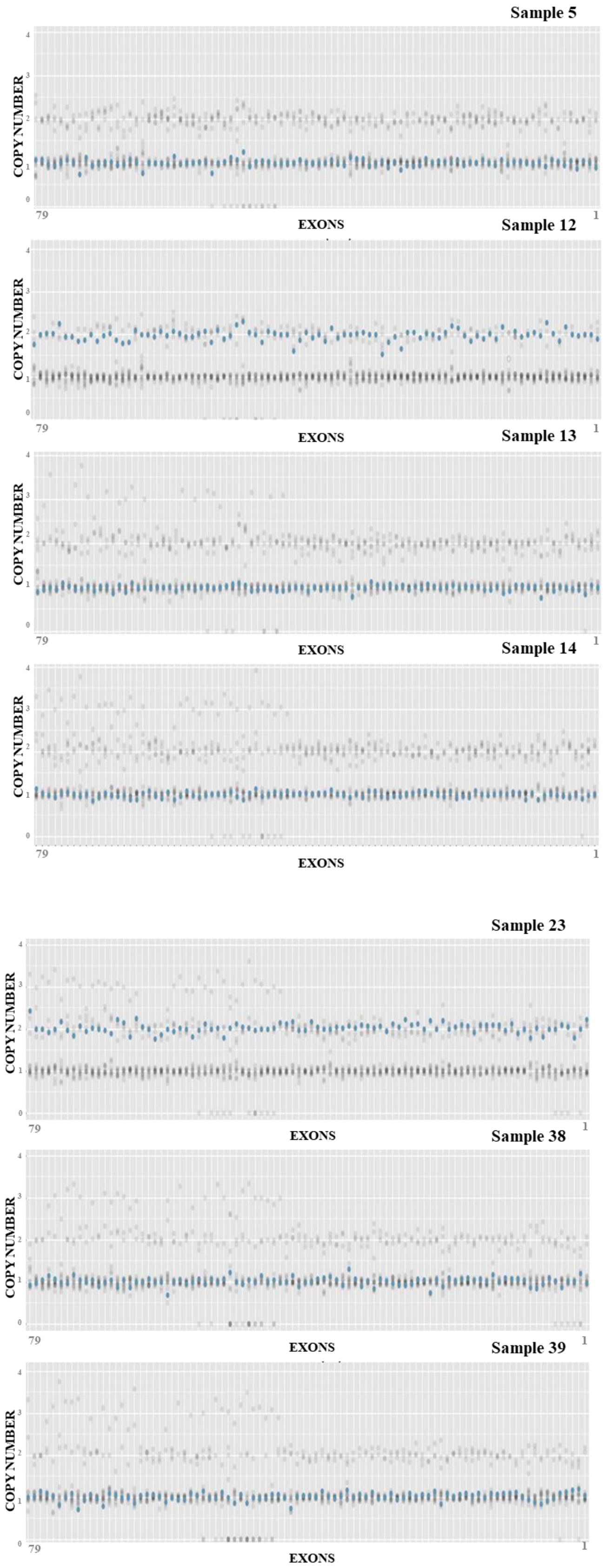

Figure S5. Panel of wild-type samples for CNV analysis. Seven samples resulted to carry no *DMD* CNVs, so showing normal profiles (blue dots).
